# Reply to Seafarers’ Occupational Noise Exposure and Cardiovascular Risk. Comments to Bolm-Audorff, U.; et al. Occupational Noise and Hypertension Risk: A Systematic Review and Meta-Analysis. *Int. J. Environ. Res. Public Health* 2020, *17*, 6281

**DOI:** 10.3390/ijerph18031188

**Published:** 2021-01-29

**Authors:** Ulrich Bolm-Audorff, Janice Hegewald, Anna Pretzsch, Alice Freiberg, Albert Nienhaus, Andreas Seidler

**Affiliations:** 1 Division of Occupational Health, Department of Occupational Safety and Environment, Regional Government of South Hesse, 65197 Wiesbaden, Germany; ulrich.bolm-audorff@rpda.hessen.de; 2 Institute and Outpatient Clinic for Occupational and Social Medicine, University Medical Center Giessen, Justus-Liebig-University, 35392 Giessen, Germany; 3 Institute and Policlinic of Occupational and Social Medicine (IPAS), Faculty of Medicine, Technische Universität Dresden, 01307 Dresden, Germany; anna.pretzsch@mailbox.tu-dresden.de (A.P.); alice.freiberg@tu-dresden.de (A.F.); ArbSozPH@mailbox.tu-dresden.de (A.S.); 4 Institute of Sociology, Faculty of Behavioral and Social Sciences, Chemnitz University of Technology, Thüringer Weg 9, 09126 Chemnitz, Germany; 5 Competence Center for Epidemiology and Health Services Research for Healthcare Professionals (CVcare), University Medical Centre Hamburg-Eppendorf (UKE), 20246 Hamburg, Germany; a.nienhaus@uke.de; 6 Department of Occupational Medicine, Hazardous Substances and Public Health, Institution for Statutory Accident Insurance and Prevention in the Health and Welfare Services (BGW), 22089 Hamburg, Germany

We are grateful for the insightful comments in [1] regarding our systematic review on the relationship between occupational noise exposure and the risk of arterial hypertension [2] and the implications it may have for seafarers. We agree that the association between occupational noise exposure and the risk of coronary artery disease and myocardial infarction requires further investigation. There is already some evidence for a positive association [3,4,5,6].

We also agree that the associations between occupational noise exposure of maritime workers and the development of hypertension or other cardiovascular diseases, such as coronary heart disease, require further investigation. The fact that noise exposure is not mentioned in existing summaries of occupational cardiovascular risk factors of seafarers [7] and that we could only include one study of seafarers [8] in our systematic review suggests that occupational noise exposure is still overlooked and under-researched in this occupational group. The unique maritime working environment could also provide especially valuable information on the effects of occupational noise exposure on cardiovascular health, as seafarers experience the same environmental noise exposures during their long confinements at sea. We believe that longitudinal studies would be particularly important in this context.

To prevent not only sensorineural hearing loss but also cardiovascular diseases, such as arterial hypertension, occupational physicians and technicians in all affected industries should promote appropriate technical, organizational and personal protective measures to reduce the level of occupational noise exposure.
